# Positive Mental Health of Finnish People Living Alone: The Role of Circumstantial Factors and Leisure-Time Activities

**DOI:** 10.3390/ijerph18136735

**Published:** 2021-06-23

**Authors:** Tytti P. Pasanen, Nina Tamminen, Tuija Martelin, Pia Solin

**Affiliations:** 1 Finnish Institute for Health and Welfare, Equality, Mental Health, FI-00271 Helsinki, Finland; nina.tamminen@thl.fi (N.T.); pia.solin@thl.fi (P.S.); 2 Non-Discrimination and Gender Equality, Finnish Institute for Health and Welfare, Equality, FI-00271 Helsinki, Finland; tuija.martelin@thl.fi

**Keywords:** single occupancy households, psychological well-being, leisure time, socio-economic status, social life

## Abstract

Living alone has become more common across Europe. Past research has consistently identified living alone as a risk factor for poor mental health while evidence on the positive dimension(s) of mental health has been scarce. Positive mental health has been associated with rather stable circumstantial factors, such as socio-economic characteristics and social relationships, and day-to-day activities in the form of leisure participation, in general populations. In this study, our objective was to assess these relationships among people living alone. We specified a structural equation model in a random sample of Finnish people living alone (*n =* 884), with the circumstantial factors as (exogenous) explanatory variables, participation in various leisure activities as mediators, and positive mental health as the outcome. In the model, more frequent engagement in several leisure-time activities, including being in contact with family/friends and physical activity in nature, were positively associated with positive mental health. The circumstantial factors that most strongly explained both leisure participation and positive mental health were the number of friends, being in a relationship, and having no limiting illnesses. In conclusion, among Finnish people living alone, social and functional factors appear to be more strongly associated with leisure participation and positive mental health than socio-economic factors.

## 1. Introduction

Household sizes have dramatically decreased in the past decades, with single households comprising on average 45% of all households in Finland and 35% in other European countries [1,2]. In the European Union (EU), 24.5% of women and 18.9% of men aged 15 or more live alone in 2019 [1]. Moreover, the number of single person households increased in total by 19% between 2010 and 2019 in the European Union, and this increase was evident among both men and women, in all adult age groups [1]. Nevertheless, scientific research on the wellbeing of those living alone has been largely conducted with a focus on the elderly, ignoring the newly-changed demographics of those living alone [3].

Living alone has been found to correlate with many social and mental health issues ranging from loneliness to financial deprivation [4]. In epidemiological research on health and well-being, living alone has usually been considered a risk factor that is ‘controlled for’ and compared with being married or cohabiting with a partner. However, those who live alone form a large and diverse group of people in very different life situations [5]. Accordingly, their health concerns, behaviour and statuses are likely to vary.

Mental health is a key aspect of overall health, and it is a much broader concept than mere presence or absence of a diagnosed mental illness [6]. The term positive mental health (also referred to as mental or subjective well-being; these terms are used interchangeably here) has been used to describe the positive spectrum of mental health, encompassing affective (hedonic) well-being, a range of positive emotional states such as happiness and contentment, and having a sense of purpose in life (eudaimonic well-being; [7]). Although mental health disorders and positive mental health often overlap, they have been found to be separate dimension of overall mental health (e.g., [8]). Concerns with mental health tend to be more common in people living alone compared with those living in with other people [9] but information on positive mental health of people living alone and its correlates has been scarce [10]. This paper addresses this gap by specifically focusing on the positive mental health of people living alone.

Many established correlates of positive mental health are relatively stable, or, circumstantial [11]. These include socio-economic or demographic attributes such as being employed or highly educated [12,13]. In addition, the role of close social relationship in determining subjective well-being has been found strong [14]. The number of close friends and having a ‘significant other’, often determined by marital status, has been consistently found to correlate with higher levels of subjective well-being [15]. Besides these established circumstantial factors, the field of human-animal relations has investigated the well-being effects of having a dog. It is known that having a dog usually promotes physical activity, provides company [16], and facilitates interaction with other people in the outdoors [17]. Although the current evidence for the overall well-being effects of having a dog has been found mixed or inconsistent in general populations [16,18], it is a compelling idea that social aspects of having a dog might be more important for the well-being of people living alone. Tentative evidence based on a small sample of people living alone, however, found no support for this idea but the topic calls for replications [19].

Besides these more stable, circumstantial factors, one’s day-to-day activities can be at least, or even more relevant predictors of subjective well-being [11,20]. Leisure time, and how one spends it, is an aspect of life that people usually have a control over and which they can adapt to meet their needs more easily than the circumstantial factors [21,22]. The kind of leisure-time activities that potentially enhance well-being are numerous. For example, based on a daily diary study, Conner et al. [23] found that creative leisure-time activities promote positive affect and flourishing; similar relationships have been found on the population level in the UK, including engagement in arts actively (such drawing, playing an instrument, or singing) and passively (such as attending art exhibitions and museums) [24,25]. Likewise, taking part in religious events, community involvement and participation in voluntary activities have been found to explain higher levels of mental and subjective well-being [12,13]. Evidence is also accumulating on the benefits of visiting natural environments such as urban parks and forests for mood and longer-term well-being [26,27]. Overall, conducting any enjoyable leisure activities have been found to correlate with life satisfaction and positive mood states in the general population [28].

Participation in enjoyable leisure-time activities is, however, affected by a variety of socio-demographic, individual and structural factors [29]. Although people living alone might have more control over their leisure time compared with those living with their spouse and/or children [30], they are known to have more health issues and financial constraints [4,31] which can hinder participation in health-promoting leisure activities [20]. For example, visiting natural environments infrequently is more common in those who do not live with a partner (based in their marital status) in the UK [32] and among the older age groups of people living alone in Finland [33].

**Figure 1:**
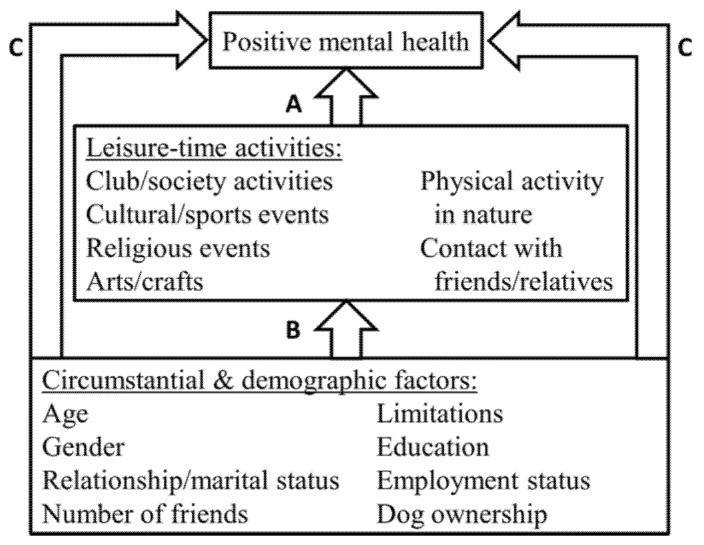
Conceptual model to assess the research questions 1–3.

In this paper, our aim is to observe the kind of circumstantial factors that play a role in leisure participation of people living alone and to evaluate to which extent they reflect on their positive mental health. We test a conceptual model illustrated in Figure 1, with focus on the following questions:

(1) Which leisure-time activities are associated with positive mental health of people living alone (paths A)?
(2) Which circumstantial factors are associated with participation in different leisure-time activities (paths B)?
(3) Which circumstantial factors are associated with positive mental health of people living alone directly (C), indirectly via leisure activities (A × B), or as their combination (C + A × B)?

By examining these questions we can obtain important information on the interplay between circumstantial factors and leisure-time activities in determining the mental well-being of people living alone, a growing demographic group known to be susceptible to a range of health and mental health issues.

## 2. Materials and Methods

### 2.1. Data

The survey, as part of the “*Positive mental health, quality of life and social support experienced by people living alone in Finland*” project, was sent to a simply-drawn random sample of 3000 Finnish residents who were officially registered as living in a one-person household from the official population registry, in autumn 2019. In the official population register one can only have a single address and hence, the sample may have included parents whose under-aged children live with them part-time or married individuals whose partner was registered in another address (such as an institution or another apartment). The sample size of 3000 was determined with the assumption of obtaining a response rate of 34.2% [34] that would result in 1026 respondents, from which we could yield population-level estimates with 95% confidence level and 3% margin of error [35].

The paper questionnaire was accompanied with information about the study, contact details of the leading investigators, and a link to the online version of the questionnaire. One reminder was sent. On total, 911 completed questionnaires were returned, of which 27 were excluded due to duplicate responses (*n =* 5), the person living with someone most of the time (*n =* 17), someone responding on behalf of the recipient (*n =* 3), and inappropriate response style (*n =* 2). The final sample, thus, consisted of *n =* 884 individuals (response rate 28%); distributions shown in Table 1. Females and older age groups were overrepresented, and sample weights, adjusting their distributions to those in the population of people living alone in Finland in the end of 2019 [2], were used in all analyses.

### 2.2. Measures

#### 2.2.1. Outcome

*Positive mental health* was measured with the 14-item Warwick-Edinburg Mental Well-Being Scale (WEMWBS; [36,37]), which has been validated and widely used in several countries [38]. The items measure aspects of eudaimonic, hedonic and social well-being experienced in the past two weeks. Each item is rated on a 5-point scale, ranging from 1 “Not at all” to 5 “All of the time”.

#### 2.2.2. Leisure-Time Activities

Leisure-time activity was enquired with a question “How often do you practice the following activities on an average?” with response options 1 “Every day or during most days”, 2 “Once or twice a week”, 3 “Once or twice a month”, 4 “Once or a few times a year”, and 5 “Less frequently or never”. The rated activities were “*club or society activities* (including posts of trust in society)”, “theatre, movies, concerts, art exhibitions, sport competitions etc.” (*cultural/sports events*), “church or other religious activities” (*religious events*), and “handicrafts, playing music, singing, photographing, painting, collecting (e.g., stamps)” (*arts/crafts*).

Physical activity in nature was enquired with the question “How often do you conduct physical activity in natural environments?” with the same response categories as above. The questionnaire series on leisure-time activities also enquired about the frequency of exercise or outdoor recreation (hunting, fishing, gardening etc.) but this item was omitted due to considerable overlap with the question regarding physical activity in nature.

The frequency of being in contact with friends or relatives was measured by asking how often the respondents are in contact with their friends or relatives in person, by phone, and via the internet. The responses were merged into being in contact via any means at least “daily or almost daily”, “1−3 times as week”, “1−3 times a month”, “less than monthly”, and “never”. Due to only a few responses to the category “never”, this category was combined with the option “less than monthly”. In line with the other leisure-time activities, these questions measured active forms of social engagement, which would presumably occur during leisure-time.

#### 2.2.3. Circumstantial Factors

Employment status was assessed in three categories: in employment/studying, unemployed (including temporarily laid-off), and retired/other. Highest level of obtained education was likewise divided into three categories: comprehensive, upper secondary and higher. Due to small number of individuals that were married or cohabiting by their marital status, marital and relationship status were combined so that all participants in a steady relationship formed one category (married/cohabiting/in a relationship), and the rest were categorised based on their marital status into single, widowed, or divorced. Having a limiting illness was assessed with the GALI instrument, asking whether the respondent has been limited because of a health problem in activities people usually do in the past six months, which has shown a good concurrent and predictive validity [39]. Dog ownership was determined based on the question “Do you have any pets?” in which one response category was “Yes, a dog”. All other (non-missing) responses were recoded as “No”. The number of friends, as an indicator of non-romantic social relationships, was assessed with the question “Do you currently have a close friend with whom you can talk confidentially about almost any issues concerning yourself?”, with the options categorised into none (“I don’t have any close friends”), one (“I have one close friend”), and two or more (“I have two close friends” or “I have several close friends”).

#### 2.2.4. Demographic Covariates

Gender was specified binary (male/female) due to a low number of responses (<5) in the ‘other’ category. Following findings showing a U-shaped curve of age on subjective well-being [12], age was specified by linear and quadratic terms. These were not the primary interest of this study but they have been found important determinants of health and subjective well-being among people living alone and thus, they were important to control for [3,5].

### 2.3. Analytical Approach

We tested a comprehensive latent variable model to examine our research questions using Mplus version 8.5 [40]. Positive mental health (WEMWBS) was specified as a latent factor and all other variables, each measuring a different underlying concept, were retained as observed/single indicator items. The WEMWBS indicators (items) and all leisure-time activities were specified as ordered categorical variables. Accordingly, we used the robust diagonally-weighed least squares (WLSMV) estimator with the recommended theta parameterisation [40]. Age was centered due to the use of quadratic term and divided by 10 due to a too large variance otherwise; hence, the estimates for a one unit change in age refer to 10 years of age.

Model fit was examined with the fit information available in WLSMV estimation, with following criteria: the χ² test/the Satorra-Bentler -corrected χ² difference test for nested models with *p* > 0.05 as indicative of good model fit, Root Mean-Square Error of Approximation (RMSEA) ≤ 0.05/0.08, Comparative Fit Index (CFI) ≥ 0.95/0.90, the Tucker-Lewis fit Index (TLI) ≥ 0.95/0.90, and Standardised Root Mean Square Residual (SRMR) ≤ 0.08 [41,42,43]. In addition, large correlation residuals were inspected with the rule-of-thumb criteria of >|0.10| [44]. As the sample was collected for a larger research project, no power analyses specific to the present study were conducted a priori. Although the sample size clearly exceeded the average sample size of approximately 200 cases in structural equation models in different disciplines (albeit critisised for being too small [44]), we placed extra attention on evaluating the overall model fit and the plausibility of the estimated parameters which could be signals of an underpowered model.

In the model specification, residual variances of all leisure-time activities were free to correlate, and all covariates explained PMH and all leisure-time activities. In interpretation, we report and assess estimates that are standardised with the option “stdy” which standardises all outcomes, including ordinal mediators, but not covariates [40]. However, to compare effect sizes of the direct (RQ1) and total effects (RQ3), the difference between the estimates was only available for the unstandardised estimates, using the ‘model constraint’ command in Mplus. Although “statistical significance” was determined at the usual criteria of *p* < 0.05, we aim to address some of the critique concerning over-emphasising *p*-values in applied research (e.g., [45]) by placing more focus on effect sizes (the standardised parameter estimates) and their relative differences rather than *p*-values. For example, in our attempt to address this issue, when discussing the results and drawing conclusions, we place more focus on the associations with the largest estimates instead of highlighting every single parameter with *p* < 0.05 (or, deeming every parameter with *p* > 0.05 as “not statistically significant”), and compare the size of the associations to each other, in addition to testing whether they differ from 0 (that is, the usual null hypothesis for each estimate) [45].

Because the leisure-time activities were specified as mediators between the circumstantial and demographic factors, the potential relationships to assess between the circumstantial factors and positive mental health include a) direct effects (individual paths C in Figure 1), controlling for all other relationships in the model b) indirect effects via each leisure-time activity (all combinations of paths A×B) c) total indirect effects (the sum of all indirect effects from a specific circumstantial factor) d) total effects (sum of the direct and total indirect effects). In this study, we were more interested in the direct, total indirect and total effects than in the numerous individual indirect connections. It is also important to note that the term ‘effect’ does not refer to causal relationships with the use of cross-sectional data, although it is commonly used within the mediation methodology literature.

### 2.4. Sensitivity Analyses

The preliminary models showed several large (>|0.10|) correlation residuals between items in WEMWBS (provided in Appendix A Table A1; similar findings as [46]). Of these, those that were conceptually similar were freed iteratively one at a time, starting from the largest residual, until all residuals were below this threshold. These post-hoc modifications resulted in improved model fit but affected the parameter estimates only trivially, and therefore the model reported as the main model constrains all of these residual correlations to 0. For the specification of age, we also tested a linear and categorical (grouped into 18–29, 30–64, and 65+) specifications but the models showed worse fit and lower variances explained and hence, we decided to retain and report the quadratic model.

## 3. Results

### 3.1. Model Fit

**Figure 2:**
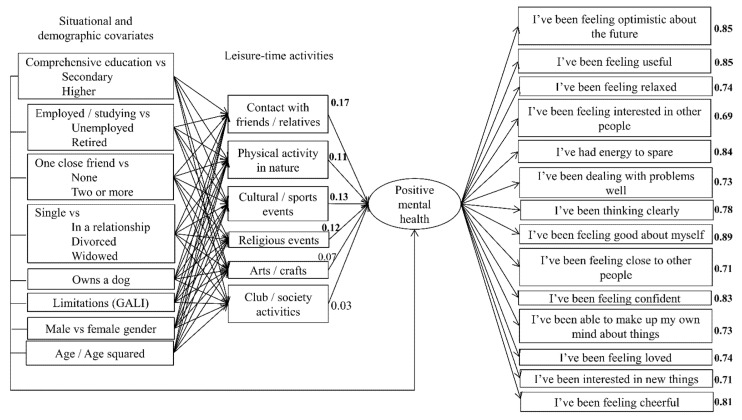
Illustration of the main reported structural equation model (*n* = 801) where positive mental health is specified as a latent factor whose loadings (reflecting correlation between the factor and the items) are shown at the right side of each item. The residual correlations between the leisure-time activities and the path estimates from each situational and demographic covariate to each leisure-time activity and positive mental health were estimated but they are not shown for clarity (provided in Table 2, Figure 3, and Appendix A Table A2). In bold: the 95% CI of the standardised estimate does not overlap with 0.

Apart from the χ² test (χ² = 1243.4, df = 337, *p* < 0.0001), the hypothesized model showed adequate fit with the data (RMSEA = 0.058, CFI = 0.940, TLI = 0.916, SRMR = 0.054). The structure of the model, including factor loadings and path estimates between leisure-time activities and positive mental health, is presented in Figure 2 (for clarity, the residual correlations between the leisure-time activities and path estimates from situational and demographic covariates are not shown in the figure but they are presented in the following sections). Many correlation residuals between the items of positive mental health exceeded the rule-of-thumb of >|0.10| (see Appendix A Table A1). Freeing the largest nine correlation residuals one at a time changed the estimates in the model only trivially and hence, we retained the original model for more detailed inspection.

Overall, the model explained substantial share of positive mental health (R^2^ = 0.37). Of leisure-time activities, the demographic and circumstantial factors best explained attending cultural and sports events (R^2^ = 0.25), followed by being in contact with friends/relatives (R^2^ = 0.25), physical activity in nature (R^2^ = 0.22), arts/crafts (R^2^ = 0.19), religious events (R^2^ = 0.17), and club/society activities (R^2^ = 0.08).

### 3.2. Research Question 1: Paths from Leisure-Time Activities to Positive Mental Health

The frequency of being in touch with friends or family, conducting physical activity in natural environments, attending sports and cultural events or religious events were all positively associated with positive mental health (Table 2). The estimates for arts and crafts, and participation in activities provided by organisations and clubs were also positive but closer to 0. The differences between the (unstandardised) estimates, however, were close to 0, suggesting that none of the specific leisure-time activities was more strongly associated with positive mental health than another.

### 3.3. Research Question 2: Paths from Circumstantial Factors to Leisure-Time Activities

Having completed higher than comprehensive education was associated with more frequent attendance of cultural or sports events (β = 0.24, s.e. = 0.12, *p* = 0.04. for upper secondary and β = 0.53, s.e. = 0.11, *p* < 0.001 for higher education) and organisations or club activities (β = 0.48, s.e. = 0.13, *p* < 0.001 and β = 0.43, s.e. = 0.12, *p* < 0.001, respectively).

Compared with those who were employed or studying, unemployed respondents attended religious events (β = 0.32, s.e. = 0.16, *p =* 0.05) and participated in clubs or organisations (β = 0.04, s.e. = 0.18, *p =* 0.024) more frequently, and conducted PA in nature (β = −0.31, s.e. = 0.14, *p =* 0.027) and attended cultural or sports events (β = −0.45, s.e. = 0.14, *p =* 0.002) less frequently. Retired respondents reported more frequent engagement in PA in nature (β = 0.29, s.e. = 0.14, *p =* 0.003), art and crafts (β = 0.42, s.e. = 0.14, *p =* 0.003), and organisation or club activities (β = 0.42, s.e. = 0.16, *p =* 0.008), compared with the employed and students.

As expected, having no close friends, compared with one, was associated with less frequent contact with family and friends (β = −0.59, s.e. = 0.13, *p* < 0.001) but not to any other leisure-time activities. Having two or more close friends, compared with one, was associated with more frequent contact with family and friends (β = 0.39, s.e. = 0.10, *p* < 0.001), conducting physical activity in nature (β = 0.24, s.e. = 0.08, *p =* 0.005), attending cultural or sports events (β = 0.20, s.e. = 0.09, *p =* 0.029), and arts/crafts (β = 0.22, s.e. = 0.09, *p =* 0.018).

Marital/relationship status was not associated with most activities. The only exception was participation in organisations or clubs, which was more frequent among those who were widowed (β = 0.28, s.e. = 0.14, *p =* 0.046) compared with those who were single.

Dog-owners engaged more frequently in physical activity in nature (β = 0.90, s.e. = 0.12, *p* < 0.001) and in arts/crafts (β = 0.47, s.e. = 0.14, *p* = 0.001) but attended less frequently cultural/sports events (β = −0.27, s.e. = 12, *p* = 0.032) and religious events (β = *−*0.31, s.e. = 0.14, *p* = 0.028) than those who did not own a dog (Table 3).

Limiting health conditions in the past six months were associated with less frequent engagement in physical activity in nature (β = *−*0.25, s.e. = 0.08, *p* = 0.003), cultural or sports events (β = −0.29, s.e. = 0.09, *p* = 0.001), and arts/crafts (β = −0.49, s.e. = 0.09, *p* < 0.001).

### 3.4. Research Question 3: Direct and Indirect Paths from Circumstantial Factors to Positive Mental Health

**Figure 3:**
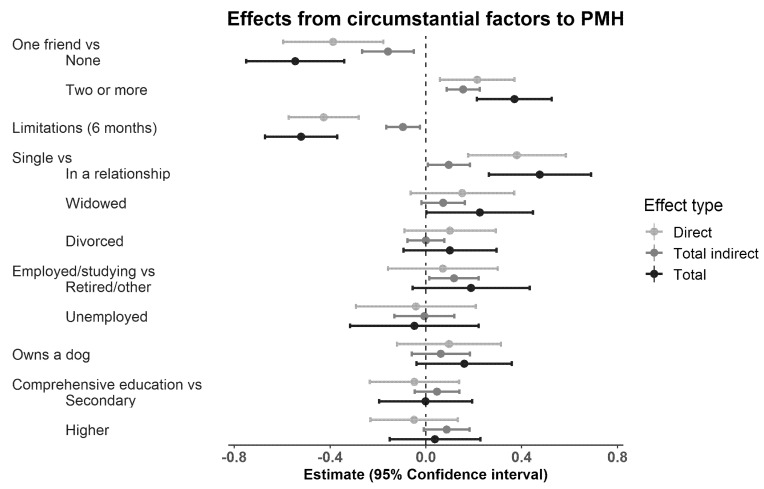
Direct, total indirect and total standardised effects from the circumstantial factors to positive mental health in the main model (*n* = 801).

Positive mental health was directly positively associated with having two or more (compared with one) friends (β = 0.21, s.e. = 0.08, *p =* 0.007; Figure 3) and being in a relationship versus being single (β = 0.38, s.e. = 0.10, *p* < 0.001), and negatively associated with having no friends (compared with one; β = −0.39, s.e. = 0.11, *p* < 0.001) and having limiting illness in the past six months (β = −0.43, s.e. = 0.08, *p* < 0.001).

Positive mental health was indirectly, via leisure participation, positively associated with having two or more (compared with one) friends (β = 0.16; Figure 3; Appendix A Table A2), being in a relationship versus being single (β = 0.10), and being retired versus employed or studying (β = 0.12), and negatively associated with having no friends (compared with one; β = −0.16), and having had a limiting illness in the past six months (β = −0.10).

The abovementioned direct and total indirect connections from circumstantial factors to positive mental health were without exceptions to the same direction (Figure 3), and therefore their combinations, that is, total effects, were also to the same direction (see Appendix A Table A2 for the exact estimates). The strongest circumstantial correlates of positive mental health were having no friends (compared with one) or limiting illnesses in the past six months and having a significant other (being in a relationship, married or cohabiting). The absolute values of their estimates were larger than those of other forms of marital status (widowed or divorced vs. single), education (both levels), unemployment (vs. employment/studentship), and dog ownership (Appendix A Table A3).

## 4. Discussion

### 4.1. Main Findings

Our study assessed the associations between circumstantial factors, leisure-time activities and positive mental health in people living alone. We found that more frequent engagement in several leisure-time activities, including being in contact with family and friends, physical activity in nature, and attending cultural/sports or religious events, were positively associated with positive mental health. The circumstantial factors that most strongly explained both leisure participation and positive mental health were the number of friends, being in a relationship, and having no limiting illnesses. We found a clear pattern showing that those who have one or more close friends and a ‘significant other’ not only have greater levels of positive mental but they also participate more often in leisure-time activities which, in turn, correlates with greater positive mental health.

Regarding our first research question, the particular leisure-time activities that were associated with greater positive mental health among people living alone were mostly the same as review studies, based on whole adult populations, have found. The beneficial effects of being in contact with friends and family, attending religious events, and visiting natural environments have been systematically registered in different populations [12,13,47] and people living alone do not appear to differ from the general populations in these regards. In line with the current evidence was also the result that the association between passive involvement in arts–such as going to the theatre and movies–and positive mental health was positive, but contrary to expectations, active forms of art involvement such singing, drawing or playing an instrument showed a much weaker connection [24,25]. Likewise, it was unexpected that participation in clubs and societies, including volunteer work, showed an inconclusive association with positive mental health. This result is contrary to prior studies in general populations [12] and tentative evidence on Finnish people living alone (in the Lapland region [48]). The underlying reasons for these unanticipated results might benefit from a more detailed examination of the specific activities within the rather broad range of activities covered in the items for arts/crafts and participation in clubs and societies. Nevertheless, when comparing the strength of the leisure-time activities associations with positive mental health, none of them stood out as more important than another, and in this regard our study concurs with that of Pressman et al. [28] on the overall importance of enjoyable leisure-time for mental well-being.

The circumstantial factors’ associations with leisure participation, assessed in our second research question, were likewise mostly to the expected direction. Consistent with earlier findings in the general populations, less frequent leisure participation was more common within those who had had limitations in the past six months [20] and those with no friends, whereas more frequent participation was more common within people living alone who had at least two friends and higher than a comprehensive education [29]. Dog ownership, however, showed a more complex pattern. Dog owners engaged more frequently in physical activity in nature (in line with [49,50]) and in arts/crafts but they attended less frequently religious, cultural, and sports events. This could be a question of trade-offs that having a dog commonly entails: while having a dog facilitates social interaction with other people and promotes health behavior in the form of physical activity [16], dogs require care and presence at home which complicate participation in other, potentially health-enhancing, leisure-time activities. Another complex relationship was that between leisure participation and employment status. Whether one is employed, unemployed or retired can pose time-related, financial, and functional constraints on leisure participation [29]. Accordingly, in our study, the retired participated in several leisure activities more often than those who were employed or studying, whereas the unemployed reported both more and less frequent participation, depending on the activity. For example, the unemployed respondents reported less frequent physical activity in natural settings, compared with the employed. Similar findings have been documented in the general Finnish adult population [33], whereas in the UK this relationship seems the other way around [32]. Given that in Finland natural settings are free to visit and relatively well accessible across the country [33], exploration on the kind of constraints that hinder engagement in physical activity in nature within unemployed people who live alone are encouraged.

Our third research question dealt with the circumstantial factors’ relationship with positive mental health. We found that the same circumstantial factors that were consistently associated with leisure participation were almost without exception also directly associated with positive mental health. For example, those who had two or more close friends (compared with one), not only had a greater level of positive mental health regardless of leisure participation, but they also participated more frequently in several leisure-time activities that were, in turn, associated with greater positive mental health. Furthermore, these associations were mostly in line with studies on general populations. The strongest circumstantial correlates of positive mental health were related to social relationships (either romantic partnership or friendships; in line with [12,13,14,15]) and functional capacity [39]. Contrary to expected (based on [12,13]), socioeconomic indicators, education and employment status, did not show a consistent association with positive mental health in our analysis. Previous studies have found socio-economic indicators to better explain low rather than high levels of positive mental health [51], which could be the reason why these socio-economic characteristics showed an inconclusive association in our study where positive mental health was assessed as a linear continuum. In the case of unemployment, which usually predicts lower subjective well-being when compared to the employed or students [12], this “null effect” could also be partly due to its mixed associations with different types of leisure participation. Similarly, dog-owners participated in some activities less and in others more frequently than those without dogs, resulting in an inconclusive association with positive mental health. This also concurs with previous studies for the general adult populations [16,18] and evidence from those living alone [19].

A major contribution of our study was the use of a methodology that enabled simultaneous assessment of a range of direct and indirect relationships between circumstantial and demographic factors and positive mental health. With a structural equation model, we could examine in detail subgroups within people living alone that not only have lower levels of positive mental health but also lower participation in leisure-time activities. Another added value of our study was the assessment of a range of leisure-time activities that are often examined in different fields. For example, although people-environment studies have reached a consensus that exposure to natural settings is beneficial for mental well-being [47], the relative importance of nature visits compared with other pleasant leisure-time activities has been yet to assess.

### 4.2. Limitations

This study had several limitations. First, the list of assessed leisure-time activities was not exhaustive of all potentially enjoyable or health-enhancing activities. Especially the amount of all leisure-time physical activity would have been useful for effect size comparison and to exclude potential confounding with physical activity in nature. Some of the direct associations between circumstantial factors and positive mental health could be explained by participation in leisure-activities other than those that we measured. Second, the study population was refined to Finnish residents living alone, and any associations found might be different in other countries with different demographic structure, cultural norms, and facilities. Third, although the response rate was comparable to other survey studies in the past 15 years [34], and we weighed the sample in terms of age and gender, it is possible that healthier and more active people were overrepresented (e.g., [52]). Fourth, we cannot infer causal relations based on our cross-sectional data. In many cases, it is likely that the present associations are bi-directional. As an example, participating more frequently in activities where one meets other people can facilitate the development of friendships, but participation might also be easier to begin and maintain for those who already have wider social networks. Fifth, it is possible that age and gender are not only associated with the level of positive mental health and leisure participation but that they also moderate these associations. For example, some forms of leisure participation might be more beneficial for males than females in terms of positive mental health. The question of age/gender moderation is outside the scope of this study but worthy of examining, for example, to target interventions on well-being promotion appropriately. Hence, this and the other limitations are all issues that we encourage prospective studies to address in future investigations.

## 5. Conclusions

Among Finnish people living alone, close social relationships and having no limiting illnesses appear to be more strongly associated with leisure participation and positive mental health than socio-economic factors or having a dog. In terms of leisure participation, contact-keeping with friends or family is equally strongly associated with positive mental health as many other activities, including physical activity in natural environments and attending cultural/sports and religious events. As studies looking at positive mental health in the population of single occupancy households have been scarce [10], replications in Finland and in other countries are needed to verify these relationships. Given the increased rates of people living alone around Europe [1] and their greater prevalence of mental health issues in comparison to those not living alone [9], the mental well-being of people living alone and its promotion is an increasingly-important public health matter.

## Figures and Tables

**Table 1:** Distributions of the study variables.

Variable Type	Variable	(Category)	*n*	% or Mean (SD)
Demographic	Gender (*n =* 882)	Female	469	53%
		Male	413	47%
	Age (*n =* 884)		884	53.9 (21.1)
Circumstantial	Education (*n =* 876)	Comprehensive	186	21%
		Upper secondary	329	37%
		Higher	362	41%
	Employment status (*n =* 870)	Employed/studying	435	50%
		Unemployed	114	13%
		Retired/other	321	37%
	Number of close friends (*n =* 870)	None	137	16%
		One	165	19%
		Two or more	569	65%
	Marital/relationship status (*n =* 860)	Single	331	39%
		Divorced	185	21%
		Widowed	141	16%
		In a relationship/cohabiting/married	203	24%
	Owns a dog (*n =* 869)		89	10%
	Limitations in the past 6 months (*n =* 868)		371	43%
Leisure-time activities	Club or society activities (*n =* 842)	Every day or most days	21	3%
		1–2 times/week	114	14%
		1–2 times/month	109	13%
		Yearly	122	14%
		Less frequently/never	476	57%
	Cultural/sports events (*n =* 854)	Every day or most days	6	1%
		1–2 times/week	35	4%
		1–2 times/month	255	30%
		Yearly	393	46%
		Less frequently/never	165	19%
	Religious events (*n =* 847)	Every day or most days	6	1%
		1–2 times/week	35	4%
		1–2 times/month	54	6%
		Yearly	221	26%
		Less frequently/never	531	63%
	Arts/crafts (*n =* 855)	Every day or most days	167	20%
		1–2 times/week	185	22%
		1–2 times/month	157	18%
		Yearly	162	19%
		Less frequently/never	183	21%
	Physical activity in nature (*n =* 871)	Every day or most days	280	32%
		1–2 times/week	294	34%
		1–2 times/month	154	18%
		Yearly	81	9%
		Less frequently/never	62	7%
	Contact with friends/relatives (*n =* 812)	Every day or most days	508	63%
		1–2 times/week	224	28%
		1–2 times/month	58	7%
		Less frequently/never	21	2%
Outcome	Positive mental health (WEMWBS score) ^1^		840	49.4 (9.7)

^1^ Sum of the 14 items; in the main analysis this was operationalised as a latent factor.

**Table 2:** Direct standardised paths from leisure activities to positive mental health in the main model (*n* = 801), controlling for all circumstantial factors (in Table 3).

		Positive Mental Health			*p*
		β	s.e.	95% CI	
Contact with friends/relatives		0.17	0.045	[0.08; 0.26]	<0.001
Physical activity in nature		0.11	0.054	[0.001; 0.21]	0.048
Cultural/sports events		0.13	0.042	[0.04; 0.21]	0.002
Religious events		0.12	0.043	[0.04; 0.21]	0.004
Arts/crafts		0.07	0.052	[−0.03; 0.17]	0.171
Club/society activities		0.03	0.043	[−0.05; 0.12]	0.46

**Table 3:** Standardised coefficients and their 95% CIs from circumstantial factors to leisure activities in the main model (*n* = 801).

	Contact with Friends/ Relatives	Physical Activity in Nature	Cultural/Sports Events	Religious Events	Arts/Crafts	Club/Society Activities
	β [95% CI]	β [95% CI]	β [95% CI]	β [95% CI]	β [95% CI]	β [95% CI]
Comprehensive education vs.						
secondary	−0.08 [−0.35; 0.2]	0.08 [−0.12; 0.28]	0.24 [0.01; 0.46]	0.01 [−0.24; 0.27]	0.06 [−0.15; 0.28]	0.48 [0.23; 0.73]
higher	0.11 [−0.16; 0.38]	−0.08 [−0.27; 0.12]	0.53 [0.31; 0.74]	−0.13 [−0.37; 0.11]	0.19 [−0.02; 0.4]	0.43 [0.19; 0.66]
Employed/studying vs.						
unemployed	0.12 [−0.22; 0.45]	−0.31 [−0.59; −0.04]	−0.45 [−0.73; −0.16]	0.32 [0; 0.64]	0.16 [−0.14; 0.46]	0.4 [0.05; 0.74]
retired	0.02 [−0.27; 0.31]	0.29 [0.03; 0.56]	0.08 [−0.19; 0.35]	0.25 [−0.05; 0.55]	0.42 [0.14; 0.7]	0.42 [0.11; 0.73]
One close friend vs.						
none	−0.59 [−0.85; −0.33]	−0.01 [−0.23; 0.21]	−0.01 [−0.26; 0.23]	−0.37 [−0.64; −0.1]	−0.04 [−0.28; 0.2]	−0.28 [−0.57; 0]
two or more	0.39 [0.19; 0.59]	0.24 [0.07; 0.4]	0.2 [0.02; 0.37]	0.16 [−0.04; 0.35]	0.22 [0.04; 0.4]	0.19 [−0.02; 0.39]
Single vs.						
in a relationship	0.15 [−0.12; 0.42]	0.15 [−0.08; 0.38]	0.18 [−0.06; 0.42]	0.11 [−0.18; 0.39]	0.22 [−0.04; 0.48]	0.14 [−0.15; 0.43]
divorced	0.08 [−0.18; 0.33]	0.03 [−0.18; 0.23]	0.01 [−0.21; 0.23]	−0.13 [−0.36; 0.09]	−0.02 [−0.24; 0.21]	0.01 [−0.24; 0.27]
widowed	0.27 [−0.03; 0.57]	−0.05 [−0.29; 0.18]	0.06 [−0.18; 0.3]	0.13 [−0.14; 0.4]	−0.01 [−0.26; 0.25]	0.28 [0.01; 0.56]
Owns a dog	0.04 [−0.29; 0.36]	0.9 [0.66; 1.14]	−0.27 [−0.51; −0.02]	−0.31 [−0.59; −0.03]	0.47 [0.2; 0.73]	−0.02 [−0.31; 0.27]
Limitations (GALI)	0 [−0.21; 0.21]	−0.25 [−0.41; −0.08]	−0.29 [−0.47; −0.11]	0.03 [−0.17; 0.23]	−0.49 [−0.66; −0.31]	−0.03 [−0.24; 0.18]
Male gender	−0.23 [−0.43; −0.04]	−0.26 [−0.41; −0.1]	−0.22 [−0.39; −0.05]	−0.31 [−0.5; −0.12]	−0.25 [−0.42; −0.07]	−0.03 [−0.22; 0.17]
Age (in 10 years)	−0.13 [−0.21; −0.05]	0.01 [−0.06; 0.08]	−0.11 [−0.18; −0.04]	0.08 [0.01; 0.16]	−0.09 [−0.16; −0.02]	−0.01 [−0.09; 0.08]
Age squared	0.04 [0.02; 0.07]	−0.07 [−0.09; −0.05]	−0.03 [−0.05; −0.01]	0 [−0.03; 0.02]	−0.05 [−0.07; −0.03]	0.02 [−0.01; 0.04]

## Data Availability

The data presented in this study is stored at the Finnish Institute for Health and Welfare (THL), it is available for research purposes on request from Dr. Pia Solin. The data are not publicly available due to privacy restrictions.

## Appendix A

**Table A1:** Observed polychoric correlation matrix (upper diagonal) and correlation residuals (lower diagonal) in the main model.

		1	2	3	4	5	6	7	8	9	10	11	12	13	14	15	16	17	18	19	20
1	WEMWBS_1		0.73	0.60	0.42	0.69	0.53	0.51	0.71	0.46	0.58	0.48	0.53	0.54	0.63	0.09	0.16	0.22	0.21	0.19	0.15
2	WEMWBS_2	0.08		0.53	0.46	0.66	0.53	0.55	0.72	0.44	0.62	0.54	0.56	0.47	0.58	0.14	0.17	0.17	0.24	0.16	0.23
3	WEMWBS_3	0.04	−0.02			0.61	0.44	0.49	0.55	0.37	0.51	0.46	0.39	0.40	0.60	0.04	0.19	0.07	0.14	0.15	0.08
4	WEMWBS_4	−0.09	−0.05	−0.01			0.34	0.32	0.37	0.64	0.40	0.32	0.52	0.50	0.50	0.26	0.22	0.23	0.13	0.09	0.17
5	WEMWBS_5	0.04	0.02	0.07	0.02		0.49	0.54	0.69	0.46	0.59	0.44	0.45	0.58	0.60	0.12	0.17	0.13	0.32	0.32	0.16
6	WEMWBS_6	−0.01	−0.01	−0.02	−0.08	−0.04		0.63	0.60	0.34	0.53	0.52	0.33	0.42	0.44	0.03	0.16	0.09	0.15	0.10	0.11
7	WEMWBS_7	−0.08	−0.04	−0.01	**−0.14**	−0.05	**0.15**		0.64	0.34	0.59	0.67	0.39	0.44	0.54	−0.01	0.12	0.08	0.13	0.08	0.09
8	WEMWBS_8	0.01	0.03	−0.04	**−0.17**	0.01	0.03	0.01		0.42	0.75	0.54	0.50	0.50	0.65	0.07	0.11	0.16	0.20	0.19	0.17
9	WEMWBS_9	−0.07	−0.08	−0.08	**0.23**	−0.06	−0.09	**−0.13**	**−0.13**		0.43	0.29	0.68	0.44	0.49	0.14	0.17	0.18	0.06	0.04	0.27
10	WEMWBS_10	−0.06	−0.01	−0.03	−0.10	−0.04	0.01	0.01	0.07	−0.08		0.61	0.49	0.47	0.64	0.10	0.11	0.07	0.13	0.14	0.19
11	WEMWBS_11	−0.06	0.00	0.00	**−0.10**	−0.09	0.08	**0.18**	−0.03	**−0.14**	0.08		0.35	0.41	0.51	0.02	0.09	0.02	0.12	0.03	0.12
12	WEMWBS_12	−0.02	0.00	−0.08	0.09	−0.10	**−0.13**	**−0.12**	−0.10	**0.24**	−0.05	**−0.12**		0.47	0.53	0.11	0.16	0.19	0.09	0.09	0.32
13	WEMWBS_13	0.02	−0.06	−0.04	0.09	0.07	−0.02	−0.04	−0.06	0.02	−0.04	−0.03	0.02		0.53	0.13	0.15	0.15	0.20	0.16	0.08
14	WEMWBS_14	0.01	−0.04	0.07	0.02	−0.01	−0.07	−0.02	−0.01	−0.01	0.03	0.00	0.00	0.03		0.19	0.10	0.13	0.17	0.13	0.17
15	clubs/societies	−0.03	0.03	−0.06	**0.17**	0.00	−0.06	**−0.11**	−0.05	0.05	−0.01	−0.07	0.01	0.03	0.08		0.24	0.27	0.18	0.07	0.11
16	events	0.00	0.01	0.05	0.09	0.01	0.02	−0.03	−0.06	0.04	−0.05	−0.04	0.02	0.03	−0.05	0.00		0.07	0.18	0.12	0.15
17	church events	0.07	0.02	−0.06	0.11	−0.03	−0.04	−0.06	0.00	0.06	−0.08	**−0.10**	0.06	0.03	−0.02	0.00	0.00		0.16	0.06	0.10
18	arts/crafts	0.02	0.05	−0.02	−0.02	**0.13**	0.00	−0.04	0.00	−0.09	−0.05	−0.03	−0.07	0.06	−0.01	0.00	0.00	0.00		0.58	0.22
19	PA in nature	0.04	0.01	0.02	−0.03	**0.17**	−0.03	−0.06	0.02	−0.09	−0.01	−0.10	−0.05	0.04	−0.02	0.00	0.00	0.00	0.00		0.07
20	social contacts	−0.03	0.04	−0.08	0.03	−0.03	−0.05	−0.08	−0.03	**0.12**	0.00	−0.03	**0.16**	−0.07	−0.01	0.00	0.00	0.00	0.00	0.00	

**In bold**: |residual| > 0.10.

**Table A2:** Total, total indirect, and direct standardised effects [95% CIs] of all circumstantial factors on PMH in the main model (*n =* 801).

		Effect Type		
Variable	(Category)	Total Effect	Total Indirect	Direct
Owns a dog		0.16 [−0.04; 0.36]	0.06 [−0.06; 0.18]	0.1 [−0.12; 0.31]
One close friend vs.				
	none	**−0.55 [−0.75; −0.34]**	**−0.16 [−0.27; −0.05]**	**−0.39 [−0.6; −0.18]**
	two or more	**0.37 [0.21; 0.53]**	**0.16 [0.09; 0.23]**	**0.21 [0.06; 0.37]**
Male gender		**−0.23 [−0.38; −0.08]**	**−0.15 [−0.22; −0.08]**	−0.08 [−0.23; 0.07]
Limitations (GALI)		**−0.52 [−0.67; −0.37]**	**−0.1 [−0.17; −0.02]**	**−0.43 [−0.57; −0.28]**
In employment/studying vs.				
	Unemployed	−0.05 [−0.32; 0.22]	−0.01 [−0.13; 0.12]	−0.04 [−0.29; 0.21]
	Retired	0.19 [−0.06; 0.43]	**0.12 [0.02; 0.22]**	0.07 [−0.16; 0.3]
Comprehensive education vs.				
	secondary	0 [−0.2; 0.19]	0.05 [−0.05; 0.14]	−0.05 [−0.23; 0.14]
	higher	0.04 [−0.15; 0.23]	0.09 [−0.01; 0.18]	−0.05 [−0.23; 0.13]
Single vs.				
	in a relationship	**0.48 [0.26; 0.69]**	**0.1 [0.01; 0.18]**	**0.38 [0.18; 0.58]**
	divorced	0.1 [−0.09; 0.3]	0 [−0.08; 0.08]	0.1 [−0.09; 0.29]
	widowed	**0.23 [0; 0.45]**	0.07 [−0.02; 0.16]	0.15 [−0.06; 0.37]
Age (in 10 years)		0.04 [−0.02; 0.1]	**−0.03 [−0.06; 0]**	**0.07 [0.01; 0.13]**
Age squared		−0.02 [−0.04; 0]	−0.01 [−0.02; 0]	−0.01 [−0.03; 0.01]

**In bold**: 95% CI does not overlap with 0.

**Table A3:** Total unstandardized effects of circumstantial factors (excluding age) and their relative comparison.

	Variable	Est.	s.e.	*p*	Comparison ^1^
1	Owns a dog	0.26	0.166	0.117	<2, 5, 10
2	No close friends (vs. one)	−0.886	0.182	<0.001	>1, 4, 6, 7, 8, 9, 11, 12
3	Two or more close friends (vs. one)	0.601	0.137	<0.001	>6, 7, 8, 11
4	Male gender	−0.37	0.126	0.003	<2, 5
5	Limitations (in the past 6 months)	−0.847	0.138	<0.001	>1, 4, 6, 7, 8, 9, 11, 12
6	Unemployed (vs. employed/studying)	−0.078	0.223	0.727	<2, 3, 5, 10
7	Retired (vs. employed/studying)	0.308	0.203	0.13	<2, 5
8	Secondary education (vs. comprehensive)	−0.001	0.161	0.993	<2, 3, 5, 10
9	Higher education (vs. comprehensive)	0.062	0.157	0.693	<2, 3, 5, 10
10	In a relationship, married or cohabiting (vs. single)	0.775	0.187	<0.001	>1, 6, 8, 9, 11, 12
11	Divorced (vs. single)	0.164	0.161	0.308	<2, 3, 5, 10
12	Widowed (vs. single)	0.365	0.187	0.05	<2, 5, 10

^1^ Based on absolute values.
